# Whole-exome sequencing reveals a rare missense variant in *DTNA* in an Iranian pedigree with early-onset atrial fibrillation

**DOI:** 10.1186/s12872-022-02485-0

**Published:** 2022-02-11

**Authors:** Mahshid Malakootian, Masoumeh Jalilian, Samira Kalayinia, Maryam Hosseini Moghadam, Mona Heidarali, Majid Haghjoo

**Affiliations:** 1 Cardiogenetic Research Center, Rajaie Cardiovascular Medical and Research Center, Iran University of Medical Sciences, Tehran, Iran; 2 Cardiac Electrophysiology Center, Rajaie Cardiovascular Medical and Research Center, Iran University of Medical Sciences, Vali-Asr St, Hashemi-Rafsanjani Blvd, Tehran, Iran

**Keywords:** Early-onset AF, *DTNA*, Genetic testing, Cardiac arrhythmia

## Abstract

Atrial fibrillation (AF) is a morbid and heritable irregular cardiac rhythm that affects about 2%–3% of the population. Patients with early-onset AF have a strong genetic association with the disease; nonetheless, the exact underlying mechanisms need clarification. We herein present our evaluation of a 2-generation Iranian pedigree with early-onset AF. Whole-exome sequencing was applied to elucidate the genetic predisposition. Direct DNA sequencing was utilized to confirm and screen the variants in the proband and his available family members. The pathogenicity of the identified nucleotide variations was scrutinized via either segregation analysis in the family or in silico predictive software. The comprehensive variant analysis revealed a missense variant (c.G681C, p.E227D, rs1477078144) in the human α-dystrobrevin gene (*DTNA*), which is rare in genetic databases. Most in silico analyses have predicted this variant as a disease-causing variant, and the variant is co-segregated with the disease phenotype in the family. Previous studies have demonstrated the association between the *DTNA* gene and left ventricular noncompaction cardiomyopathy. Taken together, we provide the first evidence of an association between a nucleotide variation in the *DTNA* gene and early-onset AF in an Iranian family. However, the genetic testing of AF in the Iranian population is still limited. This finding not only further confirms the significant role of genetics in the incidence of early-onset AF but also expands the spectrum of the gene variations that lead to AF. Additionally, it may have further implications for the treatment and prevention of AF.

**Supplementary Information:**

The online version contains supplementary material available at 10.1186/s12872-022-02485-0.

## Introduction

Atrial fibrillation (AF) is the most common irregular cardiac rhythm in clinical practice, such that its incidence and prevalence have come to the dimension of a twenty first-century cardiovascular disorder epidemic [1, 2]. Several risk factors such as age, sex, obesity, alcohol, diabetes mellitus, valvular heart diseases, and hypertension are correlated with the incidence of AF [3]. Nonetheless, about 30% of patients with AF present with a normal heart and without any association of known cardiac pathologies or conventional risk factors [4]

Mounting evidence indicates the role of genetics as important predisposing factors in the development of AF [5, 6]. Additionally, having a positive family history increases the risk of developing early-onset AF [7–11]. In familial cases, nearly 1 in 4 patients has a first-degree relative who suffers from AF [12]. According to the Iranian Registry of Atrial Fibrillation, history of AF in a first-degree relative is positive in 15.3% of the Iranian AF population [7, 13].

Thus far, research has identified more than 100 different loci and 160 genes in association with AF [6, 14]. Furthermore, mutations in cardiac ion channels (viz, potassium, sodium, and calcium) and non-ion channel coding genes such as signaling molecules, myocardial structural proteins, and cardiac transcription factors have been linked with familial or early-onset AF [15–23].

In the present study, we investigated the genetic predisposition of a 2-generation pedigree with early-onset AF by performing whole-exome sequencing (WES), followed by direct Sanger sequencing. Herein, we report the identification of a novel mutation in the human α-dystrobrevin gene (*DTNA*), which may have caused the AF phenotype in the family.

## Material and methods

### Subjects

The present study enrolled 7 members of 1 family. Some of them clinically presented early-onset AF (age ≤ 65 y), while the others had no evidence of underlying structural or systemic diseases. The study protocol was approved by the National Institute for Medical Research Development (NIMAD, 971510) and the Ethics Committee of Rajaie Cardiovascular Medical and Research Center (RHC.AC.IR.REC.1397.088). The study was conducted in accordance with the Helsinki Declaration. All the individuals who joined the study signed written informed consent.

### DNA extraction and WES

Genomic DNAs were isolated from 200 µL of peripheral blood samples of all available family members in the pedigree utilizing a DNA extraction kit (DNPTM Kit, Iran). For WES, the SOLIDv4 platform (SureSelect X Kit, Macrogen South Korea) was applied according to the manufacturer’s instructions. Sequences acquired from WES were aligned to the GRCh37/hg19 human reference genome. Then, the WES‐extracted variants were filtered, and bioinformatics software platforms PolyPhen (http://genetics.bwh.harvard.edu/pph2/), SIFT (https://sift.bii.a-star.edu.sg/), PROVEAN (http://provean.jcvi.org/index.php), and MutationTaster (http://www.mutationtaster.org/) were applied to predict the pathogenicity of the variants. The variants were classified in keeping with the guidelines of the American College of Medical Genetics and Genomics (ACMG) [24].

### Polymerase chain reaction (PCR), primer design, and Sanger sequencing

The nucleotide variations detected by WES were confirmed by Sanger sequencing with a 3500 Genetic analyzer (Applied Biosystems, USA) in the proband and evaluated in all the individuals in the family.

Concisely, specific oligonucleotides (oligos) were designed to amplify a fragment that covered the candidate variants in the suggested genes. The sequences of all the primers are presented in Additional file 1: Table S1.

The PCR condition for amplifying the region was as follows: initial denaturation at 94 °C for 5 min, followed by 30 cycles at 94 °C for 30 s, 61 °C for 30 s [*α-dystrobrevin (DTNA*)], 59 °C for 30 s [*Nebulette (NEBL)*], 60 °C for 30 s [*Sodium Voltage-Gated Channel α Subunit 5 (SCN5A)*], and 72 °C for 30 s, with a final extension at 72 °C for 10 min. The products were electrophoresed on a 1% agarose gel, stained with ethidium bromide, and visualized under ultraviolet light. Forward or reverse primers were utilized to sequence the parts of interest.

### Bioinformatics analysis

The Gene Runner (Gene Runner 6.5.50) and PerlPrimer (PerlPrimer 1.1.21) software tools were utilized to design the primers. For the analysis of the sequencing results, the BioEdit software (BioEdit 7.2.1) was applied. The identified nucleotide transitions were investigated through the UCSC Genome Browser (https://genome.ucsc.edu) and ClinVar (www.ncbi.nlm.nih.gov/clinvar) databases. Further, in silico predictive software tools such as SIFT (https://sift.bii.a-star.edu.sg), PROVEAN (provean.jcvi.org), PolyPhen-2 (genetics.bwh.harvard.edu/pph2), and MutationTaster (www.mutationtaster.org) were applied to study the pathogenesis of the detected nucleotide variations.

The Protein Homology/analogY Recognition Engine V 2.0 (Phyre2) [25] and DNASTAR (https://www.dnastar.com) bioinformatics software tools were utilized to predict the secondary and third structures of the DTNA wild type and mutant protein.

## Results

### Clinical phenotype of the family

**Fig. 1:**
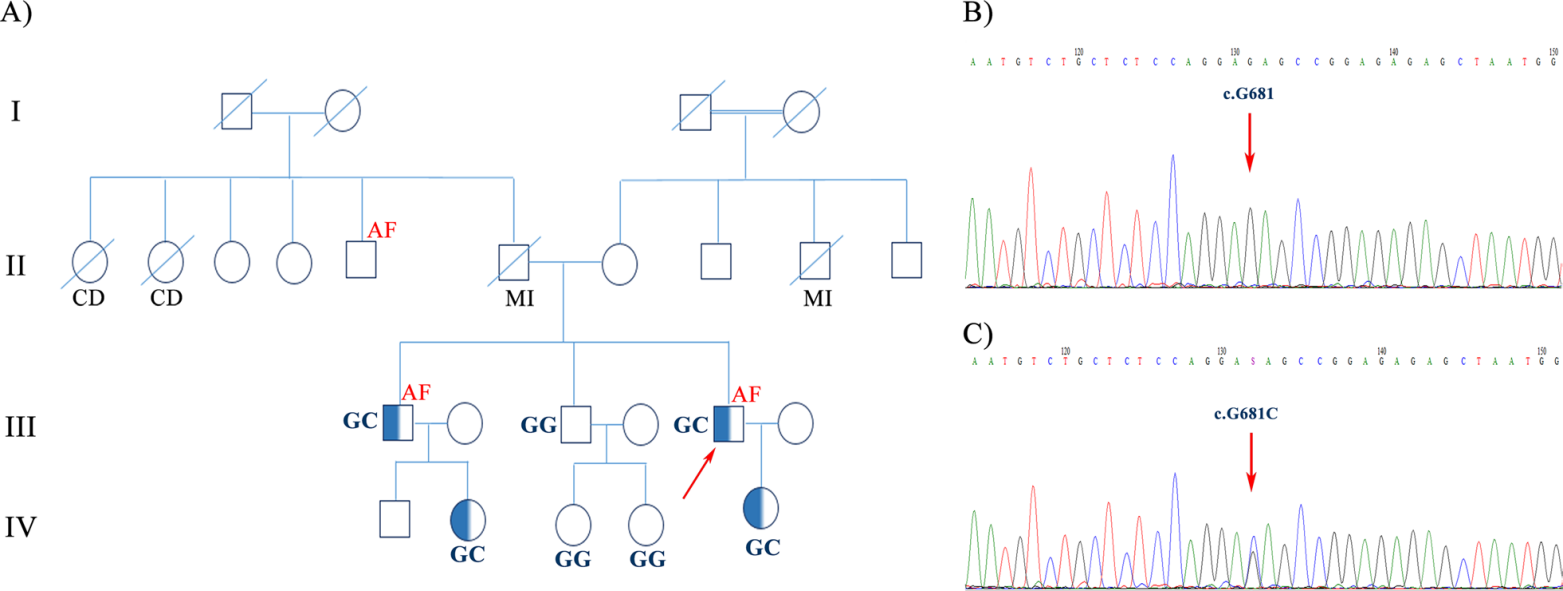
The image illustrates the pedigree, sequencing analysis, and chromatogram of the index family carrying the mutation, c.G681C, in the *DTNA* gene. **A** The pedigree of the proband revealed a positive family history of AF and MI. The genotype of the proband and his family members for *DTNA* transition demonstrated that the proband (III-5), his brother (III-1), and his brother’s children (IV-2 and IV-5) carried the c.G681C mutation of the *DTNA* gene in a heterozygote form. The other brother (III-3) and his children (IV-3 and IV-4), who were clinically normal, carried the wild type (G) of the nucleotide. **B** and **C** The chromatogram of the wild type (homozygote) and the mutant (heterozygote) of the identified transition is depicted respectively in the upper panel and the lower panel. AF: Atrial Fibrillation, MI: Myocardial Infarction

The family of interest was a 2-generation pedigree suffering from AF and heart diseases (Fig. 1).

**Fig. 2:**
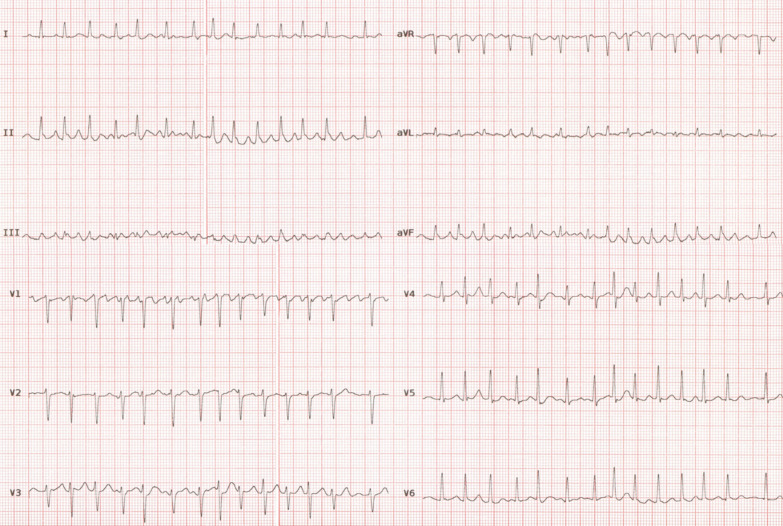
ECG of proband (III-5) shows narrow QRS tachycardia with irregular ventricular response and fibrillatory atrial activity

The proband in the family (III-5) was a 47-year-old man, who was admitted to Rajaie Cardiovascular Medical and Research Center with suspected AF. For the first time, AF was diagnosed during the evaluation of plasmacytomas. He was asymptomatic. Electrocardiography showed AF with a rapid ventricular rate (Fig. 2). The AF rhythm was observed before the commencement of chemotherapy. Transthoracic echocardiography showed a decreased left ventricular function (ejection fraction = 25%), a normal left ventricular size, mild left atrial enlargement, mild-to-moderate mitral regurgitation, no left ventricular hypertrophy, and no noncompaction pattern. No evidence of late enhancement or left ventricular noncompaction was observed in cardiac magnetic resonance imaging. AF was initially converted successfully into the sinus rhythm with direct-current cardioversion. Electrocardiography after the cardioversion showed a normal sinus rhythm with no evidence of left atrial abnormality or left ventricular hypertrophy. After 3 months, AF episodes returned. Therefore, cryoballoon pulmonary vein isolation was done, which was successful. One month after the procedure, the left ventricular function showed a significant improvement (45–50%) (Additional file 1: Table S2).

The proband’s medical history was otherwise not significant for hypertension, diabetes, high cholesterol, and other cardiovascular diseases. His family history was positive for AF in one of his brothers (III-I) and his uncle (paternal family). There was a positive history of heart diseases from both maternal and paternal sides.

The proband’s older brother (III-I) presented with drug-refractory paroxysmal AF at the age of 46 years for the first time. He also had a history of newly diagnosed systemic hypertension. Resting 12-lead electrocardiography showed a normal sinus rhythm and normal PR, QRS, and QTc intervals with no evidence of left atrial abnormality, left ventricular hypertrophy, or noncompaction patterns. Transthoracic echocardiography revealed a normal left ventricular function (55%), normal left atrial dimensions, a good valvular function, and no evidence of left ventricular hypertrophy. He underwent successful percutaneous pulmonary vein isolation using a 3D mapping system and radiofrequency energy.

At the age of 50 years, he presented with new-onset typical angina pectoris. Coronary angiography showed single-vessel disease, for which he received medical therapy. His angina pectoris progressed over time, and repeat coronary angiography when he was 55 years of age showed triple-vessel disease, for which he received 3 coronary stents. Two years after the percutaneous coronary intervention, he experienced recurrent episodes of AF; consequently, cryoballoon pulmonary vein isolation was performed, which was successful. During a 1-year follow-up after the second pulmonary vein isolation, he was in a stable medical condition (Additional file 1: Table S2).

The other brother of the proband and the other members of the proband’s family who were available for examinations were apparently normal (Additional file 1: Table S2).

### Genetic findings

For the determination of the causative genetic alterations in the family, an unbiased next-generation DNA sequencing, which covered the entire coding exons (WES), was performed. WES was accomplished with a mean target coverage rate of 100X. The results were successively analyzed with Bowtie, [26] Freebayes, [27] and SnpSift. Filtering was accomplished based on different projects (viz, db‐SNP, gnomAD v2, and 1000 Genomes Project databases) and other available population databases (eg, Iranome).

Three candidate variants were submitted for co-segregation analysis, and only 1 survived (Table 1).

**Table 1:** Information of the identified nucleotide variations of the genes in the proband of the pedigree

Gene	NM/NP	Variant	Type	RS	Location	In silico assessments					Frequency (allele count)		
						Polyphen2	Mutation taster	Provean	Sift	Clin Var	Iranome Allel frequency	gnomAD	1000 Genome
DTNA	NM_001198943.1 NP_001185872.1	c.G681C p.E227D	Het	rs1477078144	Chr 18q12.1 Exon6	Probably damaging	Disease causing	Neutral	Deleterious	NA	0	0	0
NEBL	NM_006393.3 NP_006384.1	c.T298C p.S100P	Het	-	Chr 10p12.31 Exon3	-	Disease causing	Deleterious	–	NA	0	0	0
SCN5A	NM_001099404.2 NP_001092874.1	c.A1673G p.H558R	Het	rs1805124	Chr3p22.2 Exon12	Benign	Polymorphism	Neutral	–	With Pathogenic allele	1600	3143 Het 20,427	151 Het 852

The c.G681C transition (rs1477078144) was positioned in a conserved part of exon 6 of the *DTNA* gene located on Chr 18q12.1 (the GRCh37/hg19), resulting in a Glu227-to-Asp substitution in the α-helix structure of the protein. This variant is reported neither in the 1000G Project nor in the Iranome Database. The in silico analysis of this variant via PolyPhen, SIFT, MutationTaster, and PROVEAN predicted this variant as probably damaging, deleterious, disease-causing, and neutral, respectively.

Sanger sequencing of this variant was performed in the proband and all 6 family members to confirm the presence and pattern of inheritance (Fig. 1). The Sanger sequencing upshots demonstrated that the index patient (III-5) and his affected brother (III-1) carried the variant in a heterozygous status (GC), while the other unaffected brother (III-3) and his children (IV-3 and IV-4) carried the wild type of the variant (GG) (Fig. 1).

The proband’s only child (IV-5, 16 years old) and one of the 2 children of the affected brother (IV-2, 23 years old) carried the probable pathogenic variant like their respective father in the heterozygous form (Table 2). Both children appeared normal.

**Table 2:** Genotypes of all 3 identified variants in 7 available members of the family

Individuals	DTNA(NM_001198943.1) (c.G681C)	NEBL(NM_006393.3) (c.T298C)	SCN5A(NM_001099404.2) (c.A1673G)
III-1	GC	TT	AG
III-3	GG	TT	AG
III-5	GC	TC	AG
IV-2	GC	TT	AG
IV-3	GG	TT	AG
IV-4	GG	TT	AG
IV-5	GC	TC	AG

The proband’s uncle (II-5), aged 62 years, showed symptoms of AF at an earlier age, but he was not available for the present study (Fig. 1).

The second (c.T298C) and third (c.A1673G, rs1805124) variations were harbored in exon 3 and exon 12 of the *NEBL* and *SCN1A* genes, respectively. The c.T298C transition of the *NEBL* gene caused a Ser100-to-Pro substitution, and the c.A1673G nucleotide change led to the replacement of histidine 558 with arginine of SCN1A. The c.T298C variant is reported neither in the 1000G Project nor in the Iranome Database. PolyPhen, SIFT, PROVEAN, and MutationTaster programs predicted this variant as disease-causing and deleterious. The Sanger sequencing of this variant demonstrated the heterozygote states of this variant in the proband and his son. Neither of his brothers (affected and healthy ones) and their children carried this variant (Table 2). The third candidate variant, c.A1673G, was harbored in the *SCN1A* gene and previously reported as pathogenic. The results of the direct sequencing of this variant showed the heterozygote form of this variant in the proband and all his examined family members (Table 2).

## Discussion

In recent decades, several approaches such as linkage analysis, candidate gene, and next-generation sequencing have revealed distinct disease-causing nucleotide variations in many genes linked to AF [14, 28]. Additionally, genome-wide association studies have introduced some loci associated with AF [6, 29]. Although these findings have provided fresh insights into the underlying pathophysiology of AF and may clarify novel therapeutic pathways, the vast majority of the heritability of AF remains baffling.

In this study, we found a nucleotide transition, c.G681C, in the *DTNA* gene, which probably caused early-onset AF in an autosomal dominant inheritance pattern in an index Iranian family. To the best of our knowledge, the present study is the first investigation to report the point nucleotide variation in the *DTNA* gene associated with AF. This rare variant is not reported in the 1000G Project, the gnomAD Database, and the Iranome Database. In addition, most of the in silico software tools that we employed in the current investigation predicted this variation as probably damaging, deleterious, and disease-causing.

Notably, α-dystrobrevin is related to dystrophin-related and dystrophin-associated proteins, which are thought to have a significant role in the stability and maintenance of the plasma membrane in the course of muscle contraction and relaxation, [30] while the exact function of dystrobrevin remains to be determined. A previous study reported that α-dystrobrevin encoded by the *DTNA* gene made a scaffold unit structure at the sarcolemma of the heart muscle and was engaged in maintaining the structural integrity of muscle fibers [31]. Previous studies have demonstrated that mutations in *α*-dystrobrevin lead to left ventricular noncompaction cardiomyopathy [31, 32] and Meniere’s disease [33]. In 2001, Ichidea et al. reported a 362C-T transition (heterozygote form) in exon 3 of the *DTNA*, which substituted pro121-to-leu (P121L) at a protein level as a causative nucleotide alteration and resulted in left ventricular noncompaction in 6 affected members of a 4-generation Japanese family [32]. Further, a heterozygous mutation, c.146A-G, at a position of 146 resulting in an amino acid change from asparagine to serine at codon 49 was identified in a 39-year-old man with a diagnosis of left ventricular noncompaction cardiomyopathy [31]. The functional analysis of this variation confirmed the causal role of the DTNA-p.N49 S mutation in the pathogenesis of left ventricular noncompaction cardiomyopathy by generating a transgenic mouse model expressing the DtnaN49S mutant specifically in the heart [31]. The novel heterozygous missense variant was recognized in the *DTNA* gene (chr18: 32462094G.T), causing an amino acid change (valine to phenylalanine) in a patient with autosomal-dominant familial Meniere’s disease [33]. Furthermore, 13 different variations of the *DTNA* gene in addition to the 2 aforementioned ones related to left ventricular noncompaction cardiomyopathy and Meniere’s disease have been reported with conflicting interpretations of pathogenicity according to the ClinVar Database (Additional file 1: Table S3).

Our segregation analysis demonstrated that the proband and his affected brother (III-1), together with the their respective children (IV-2 and IV-5), carried the c.G681C transition (rs1477078144) in a heterozygote status in the *DTNA* gene, while the proband’s unaffected brother (III-3) and his children (IV-3 and IV-4) did not carry the mutation and were normal based on clinical assessments as well. The children of the affected brothers in the family were apparently normal, which is probably due to their young age or the incomplete penetrance of the mutation. It is probable that the proband’s uncle (paternal side), who also had AF, carried the mutation. Unfortunately, he was not available for further analysis.

**Fig. 3:**
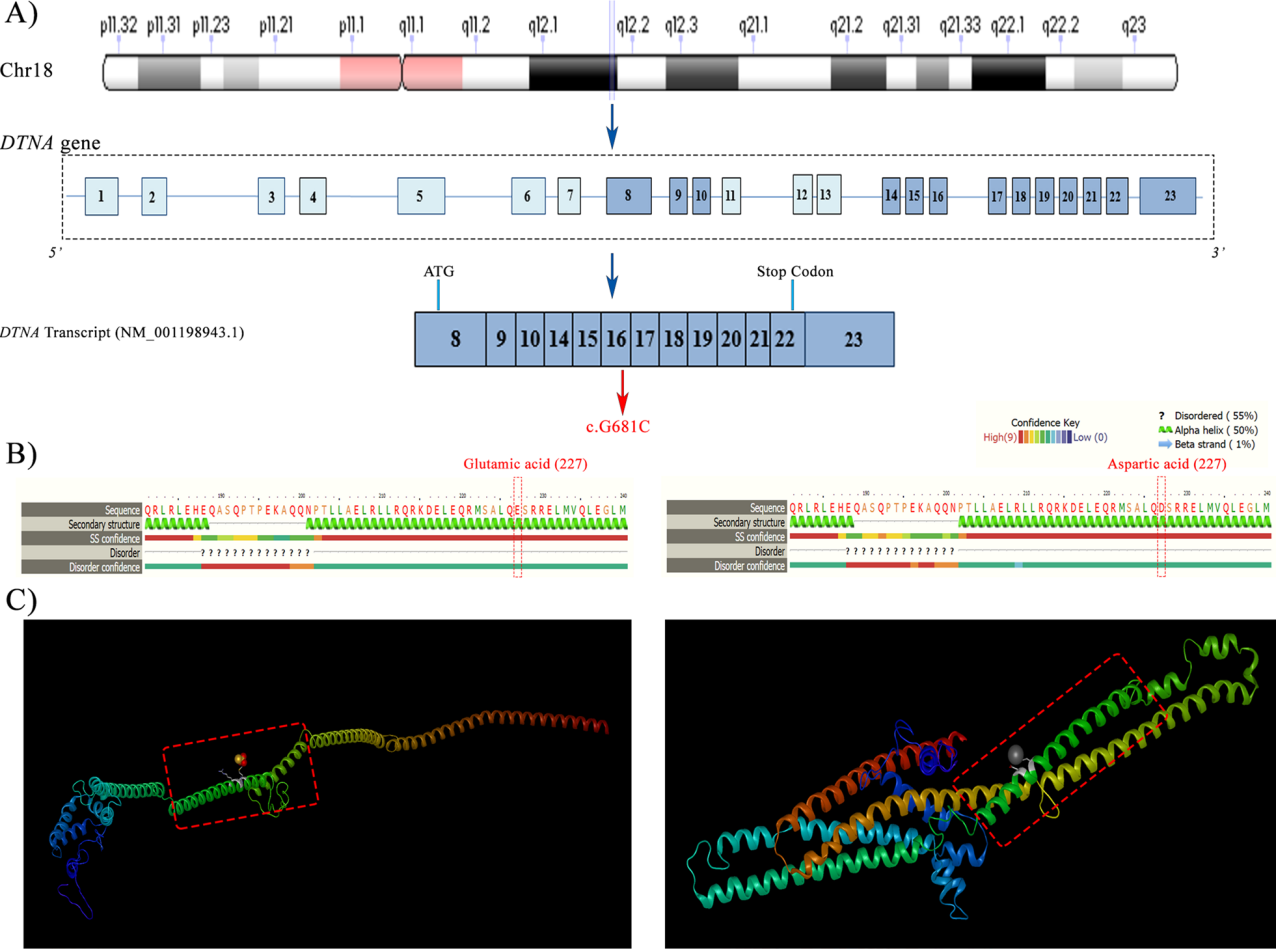
The image depicts the genomic and protein organization of DTNA. **A** The schematic view of the location of the *DTNA* gene in chr18q21.1. **B** The genetic organization of the *DTNA* gene and the transcript spliced variant of *DTNA* (NM_001198943.1), corresponding to the *DTNA* coding exon (with common colors). The mutation, located in exon 6 of the transcript, is demonstrated in red. **B** and **C** The second and third structures of the DTNA protein from Phyre2 and DNASTAR software tools, respectively. The transition replaced glutamic acid (**E**) with aspartic acid (**D**) at a position of 227 of the DTNA protein

Moreover, the whole *DTNA*, which is located in chromosome 18q12 and is composed of 23 coding exons, ranges in size from 9 to 214 bp (Fig. 3). The largest open reading frame of dystrobrevin is in a transcript, 6.5 kb in length, termed “dystrobrevin-1”, which is highly expressed in the brain and muscles. Other transcripts of *DTNA* encode the functional isoforms of dystrobrevin categorized as class I and class II [30]. The aforementioned isoforms are highly expressed in cardiac muscle as well as the brain and skeletal muscles. Class-I transcripts, characterized by alternatively spliced 3’ ends, are predicted to create truncated protein isoforms. Class-II transcripts, characterized by a different 5’ start site, are harbored in exon 8 of the *DTNA* gene [30, 34]. The mutation that we identified in the present study (c.G681C, p.E227D, rs1477078144) is harbored in a transcript with NM-001198943.1 and belongs to the class-II transcripts of *DTNA*, which is highly expressed in cardiac muscle.

According to UniProt database (https://www.uniprot.org/uniprot/Q9Y4J8#interaction) (Q9Y4J8), DTNA interacts with Alpha-catulin (CTNNAL1) which is modulate the Rho pathway signaling which has important role in cardiovascular physiology and pathophysiology [35]. It is suggested mutation in the *DTNA* gene may have disturb the interaction of the gene at the protein level and indirectly effect the Rho signaling pathway. Although more functional studies are required to delve the exact mechanism of this mutation on the function of DTNA protein.

Previous studies have demonstrated that the majority of mutations (both loss-of-function and gain-of-function types) related to AF are harbored in the genes encoding ion channel subunits [6, 15, 21–23]. However, disease-causing mutations in non-ion channel coding genes have also been reported [18–20]. In line with the latter one, in the present study, the identified mutation was located in a non-ion channel coding gene. Therefore, many more molecular genetic studies are needed to clarify new genetic alterations and validate genotype–phenotype associations with a view to shedding light on the pathophysiological pathways of AF.

### Limitations

The results of the present study should be interpreted in light of certain limitations. Functional genomic studies were not carried out in the present study. In addition, experimental studies and expression analyses are essential to determine the exact role of this variant responsible for AF. The transition was not assessed in the proband’s uncle, who suffered from AF, and nor was it evaluated in the other apparently normal family members who were not available for the study.

## Conclusions

The results of the current study present the first evidence for the association between a rare missense variant in *DTNA* and early-onset AF. Not only do these findings expand our knowledge regarding the genetics of AF but also they may have further implications for the treatment and prevention of AF. Although the discovery of novel AF-associated genetic variants has gained momentum in recent years, there is a paucity of information on the genetic analysis of AF in the Iranian population. The genetic basis of AF in the Iranian population undoubtedly needs further in-depth research.

## Supplementary Information

**Additional file 1: Table S1.** Sequences of the oligos utilized for polymerase chain reaction and Sanger sequencing. **Table S2.** Clinical information of all the available members of the pedigree. **Table S3.** All the nucleotide variations in the ClinVar Database that are submitted as pathogenic and the ones with conflicting interpretations concerning pathogenicity. As is shown in the table, except for 2 variations, c.146A > G and c.362C > T, which were causative for left ventricular noncompaction cardiomyopathy, all the other transitions have not been determined as causative variations for diseases yet. LVNC: Left Ventricular Noncompaction Cardiomyopathy, DCM: Dilated Cardiomyopathy, HCM: Hypertrophic Cardiomyopathy, MD: Meniere’s disease.

## Data Availability

*Accession number* The accession number of the reported variant in paper is available in clinVAR repository with the VCV001224303 accession number. The data sets presented in the present study can be reached in online repositories. The identified nucleotide transitions were analyzed through the UCSC Genome Browser (https://genome.ucsc.edu) and ClinVar (www.ncbi.nlm.nih.gov/clinvar) databases. In silico predictive software tools such as SIFT (https://sift.bii.a-star.edu.sg), PROVEAN (provean.jcvi.org), PolyPhen-2 (genetics.bwh.harvard.edu/pph2), and MutationTaster (www.mutationtaster.org) were applied to study the pathogenesis of the detected nucleotide variations. The Protein Homology/analogY Recognition Engine V 2.0 (Phyre2) and DNASTAR (https://www.dnastar.com) bioinformatics software tools were utilized to predict the secondary and third structures of the DTNA wild type and mutant protein. The accession number and all the repositories used for the study are mentioned in the article as well.

## Abbreviations

AF: Atrial fibrillation
*DTNA*: Human α-dystrobrevin gene
PCR: Polymerase chain reaction
Oligos: Oligonucleotides
*NEBL*: Nebulette
*SCN5A*: Sodium voltage-gated channel α subunit 5
LVNC: Left ventricular noncompaction cardiomyopathy
DCM: Dilated cardiomyopathy
HCM: Hypertrophic cardiomyopathy
MD: Meniere’s disease
