# Supplementary material for: Whole-exome sequencing reveals a rare missense variant in DTNA in an Iranian pedigree with early-onset atrial fibrillation
Source: BMC Cardiovasc Disord. 2022 Feb 11;22:37. doi: 10.1186/s12872-022-02485-0 (PMC8832862; doi:10.1186/s12872-022-02485-0)
Supplement: Supplementary file 1 — Additional file 1: Table S1. Sequences of the oligos utilized for polymerase chain reaction and Sanger sequencing. Table S2. Clinical information of all the available members of the pedigree. Table S3. All the nucleotide variations in the ClinVar Database that are submitted as pathogenic and the ones with conflicting interpretations concerning pathogenicity. As is shown in the table, except for 2 variations, c.146A > G and c.362C > T, which were causative for left ventricular noncompaction cardiomyopathy, all the other transitions have not been determined as causative variations for diseases yet. LVNC: Left Ventricular Noncompaction Cardiomyopathy, DCM: Dilated Cardiomyopathy, HCM: Hypertrophic Cardiomyopathy, MD: Meniere’s disease. [file 12872_2022_2485_MOESM1_ESM.docx]

**Additional file 1: Table S1**: Sequences of the oligos utilized for polymerase chain reaction and Sanger sequencing

| **Gene(Ref Seq)** | | **Primer Sequence (F/R)** | | **Sequencing** | **Length** |
| --- | --- | --- | --- | --- | --- |
| **DTNA-**EXON6 | NM_001198943.1 | F | ACAGTCCACAAGTCCCTCAAG | F | 295 bp |
|  |  | R | GTTGAGGGAAATCACATACAGC |  |  |
| **NEBL-**EXON3 | NM_006393.3 | F | CTTCAGAATCCTGGAGTTTGTC | F | 430 bp |
|  |  | R | GCAACTACTGACAGCACATTAATC |  |  |
| **SCN5A-**EXON12 | NM_001099404.2 | F | TCTAACCCCACATCCCCTC | F | 715 bp |
|  |  | R | TGGATGCTCTATGAGGGCC |  |  |

**Table S2.** Clinical information of all the available members of the pedigree. Abbreviations: PR=PR interval; QTc= corrected QT interval; LAA= left atrial abnormality in ECG; LVEF= left ventricular ejection fraction; HTN= systemic hypertension; CAD=coronary artery disease; PAD=peripheral artery disease. *First LVEF was 25%. Final EF after AF catheter ablation increased to 50%.

| **ID** | **Sex** | **Age (yrs)** | **Onset age (yrs)** | **PR** | **QRS**  **Morphology** | **QTc** | **LAA** | **LVEF** | **Obesity** | **HTN** | **CAD** | **PAD** | **Smoking** |
| --- | --- | --- | --- | --- | --- | --- | --- | --- | --- | --- | --- | --- | --- |
| **III-1** | M | 57 | 46 | 160 | normal | 420 | no | 50% | no | yes | yes | no | no |
| **III-3** | M | 55 | - | 140 | normal | 440 | no | 55% | no | no | no | no | no |
| **III-5** | M | 47 | 47 | 160 | normal | 420 | no | 25%, 50%* | no | no | no | no | no |
| **IV-2** | F | 23 | - | 140 | normal | 415 | no | 60% | no | no | no | no | no |
| **IV-4** | F | 19 | - | 140 | normal | 420 | no | 60% | no | no | no | no | no |
| **IV-3** | F | 26 | **-** | 140 | normal | 440 | no | 60% | no | no | no | no | no |
| **IV-5** | F | 16 | - | 150 | normal | 430 | no | 60% | no | no | no | no | no |

**Table S3.** All the nucleotide variations in the ClinVar Database that are submitted as pathogenic and the ones with conflicting interpretations concerning pathogenicity. As is shown in the table, except for 2 variations, c.146A>G and c.362C>T, which were causative for left ventricular noncompaction cardiomyopathy, all the other transitions have not been determined as causative variations for diseases yet.

LVNC: Left Ventricular Noncompaction Cardiomyopathy, DCM: Dilated Cardiomyopathy, HCM: Hypertrophic Cardiomyopathy, MD: Meniere’s disease

| **DTNA** | | | | | | |
| --- | --- | --- | --- | --- | --- | --- |
| **Number** | **Transition** | **Protein change** | **Ref Seq** | **RS** | **Condition(s)** | **Clinical significance**  **(Last reviewed)** |
| 1 | c.146A>G | p.Asn49Ser | NM_032978.7 | rs775975702 | LVNC | Pathogenic |
| 2 | c.362C>T | p.Pro121Leu | NM_032978.7 | rs104894654 | LVNC | Pathogenic |
| 3 | c.68-7G>A | - | NM_032978.7 | rs372126412 | DCM, LVNC | Conflicting interpretations of pathogenicity |
| 4 | c.54G>A | p.Leu18= | NM_032978.7 | rs754265551 | LVNC | Conflicting interpretations of pathogenicity |
| 5 | c.68-7G>A | - | NM_032978.7 | rs372126412 | LVNC, DCM | Conflicting interpretations of pathogenicity |
| 6 | c.177A>G | p.Ile59Met | NM_032978.7 | rs1057518968 | LVNC | Conflicting interpretations of pathogenicity |
| 7 | c.229A>G | p.Asn77Asp | NM_032978.7 | rs147782267 | LVNC | Conflicting interpretations of pathogenicity |
| 8 | c.537G>A | p.Thr179= | NM_032978.7 | rs11877640 | LVNC | Conflicting interpretations of pathogenicity |
| 9 | c.955A>G | p.Met319Val | NM_032978.7 | rs141981161 | LVNC | Conflicting interpretations of pathogenicity |
| 10 | c.1000G>A | p.Val334Met | NM_032978.7 | rs148123045 | LVNC | Conflicting interpretations of pathogenicity |
| 11 | c.1207C>T | p.His403Tyr | NM_032978.7 | rs139872140 | LVNC | Conflicting interpretations of pathogenicity |
| 12 | c.1228G>A | p.Asp410Asn | NM_032975.4 | rs144880521 | LVNC | Conflicting interpretations of pathogenicity |
| 13 | c.1757C>T | p.Pro586Leu | NM_032975.4 | rs145425478 | HCM | Conflicting interpretations of pathogenicity |
| 14 | c.1799G>A | p.Arg600Gln | NM_001198939.2 | rs138143719 | LVNC | Conflicting interpretations of pathogenicity |
| 15 | c.1963G>T | p.Val655Phe | NM_001198938.2 | - | MD | Conflicting interpretations of pathogenicity |
